# Automated Pre-Analytic Processing of Whole Saliva Using Magnet-Beating for Point-of-Care Protein Biomarker Analysis

**DOI:** 10.3390/mi10120833

**Published:** 2019-11-30

**Authors:** Benita Johannsen, Lara Müller, Desirée Baumgartner, Lena Karkossa, Susanna M. Früh, Nagihan Bostanci, Michal Karpíšek, Roland Zengerle, Nils Paust, Konstantinos Mitsakakis

**Affiliations:** 1 Hahn-Schickard, Georges-Koehler-Allee 103, 79110 Freiburg, Germany; lara-mueller95@gmx.de (L.M.); Desiree.Baumgartner@imtek.uni-freiburg.de (D.B.); karkossal@online.de (L.K.); Susanna.Frueh@Hahn-Schickard.de (S.M.F.); Roland.Zengerle@hahn-schickard.de (R.Z.); Nils.Paust@Hahn-Schickard.de (N.P.); 2 Laboratory for MEMS Applications, IMTEK—Department of Microsystems Engineering, University of Freiburg, Georges-Koehler-Allee 103, 79110 Freiburg, Germany; 3 Section of Periodontology and Dental Prevention, Division of Oral Diseases, Department of Dental Medicine, Karolinska Institutet, Alfred Nobels Allé 8, 14104 Huddinge, Stockholm, Sweden; nagihan.bostanci@ki.se; 4 BioVendor—Laboratorní medicína a.s., Research & Diagnostic Products Division, Karasek 1767/1, Reckovice, 62100 Brno, Czech Republic; karpisek@biovendor.com; 5 University of Veterinary and Pharmaceutical Sciences Brno, Faculty of Pharmacy, Palackeho trida 1946/1, 61242 Brno, Czech Republic

**Keywords:** point-of-care, pre-analytics, protein biomarkers, magnet-beating, whole saliva, diagnostics, immunoassay, ELISA, centrifugal microfluidics

## Abstract

Saliva offers many advantages for point-of-care (PoC) diagnostic applications due to non-invasive, easy, and cost-effective methods of collection. However, the complex matrix with its non-Newtonian behavior and high viscosity poses handling challenges. Several tedious and long pre-analytic steps, incompatible with PoC use, are required to liquefy and homogenize saliva samples before protein analysis can be performed. We apply magnet-beating to reduce hands-on time and to simplify sample preparation. A magnet in a chamber containing the whole saliva is actuated inside a centrifugal microfluidic cartridge by the interplay of centrifugal and magnetic forces. Rigorous mixing, which homogenizes the saliva sample, is then initiated. Consequently, fewer manual steps are required to introduce the whole saliva into the cartridge. After 4 min of magnet-beating, the processed sample can be used for protein analysis. The viscosity of whole saliva has been reduced from 10.4 to 2.3 mPa s. Immunoassay results after magnet-beating for three salivary periodontal markers (MMP-8, MMP-9, TIMP-1) showed a linear correlation with a slope of 0.99 when compared to results of reference method treated samples. Conclusively, magnet-beating has been shown to be a suitable method for the pre-analytic processing of whole saliva for fully automated PoC protein analysis.

## 1. Introduction

Saliva is becoming an increasingly popular sample matrix for diagnostic purposes due to its wide range of diagnostic applications including for systemic diseases, oral cancer, cardiac and cardiovascular diseases, periodontal disease (and overall oral health), as well as infectious diseases like HIV (human immunodeficiency virus) and many more [1,2,3,4,5,6,7,8]. Saliva contains around 5500 different types of proteins [9] and therefore shows great potential to be implemented in many other diagnostic applications. The collection of saliva is easy and can be done with various methods, each of which has its advantages and disadvantages but important is that the same collection method is consistently used when performing a study [10]. Any collection method is non-invasive and cost-effective, rendering whole saliva a promising sample matrix for point-of-care (PoC) applications [3,11,12,13]. It does, however, present several sample processing challenges both for manual and automated systems because of its non-Newtonian behavior [14,15], high viscosity due to the glycoprotein content, patient-dependent rheological properties [16,17], as well as variations in sample composition between stimulated and non-stimulated sample collection methods (in the former, salivary flow is mechanically or chemically increased) [18,19]. Such features may not only result in challenging sample handling (inaccurate volume metering), but also in highly demanding technical specifications for sample processing in automated systems [20,21]. Furthermore, the concentration ranges of most of the currently known salivary biomarkers that can be used for diagnostic purposes are lower than those of the same biomarkers when circulating in blood [2]. Besides, the detection of more than one salivary biomarker is required for most applications [18,22]. Thus, the sample processing challenges may result in inaccurate or irreproducible measurements of biomarker concentrations and consequently in misdiagnosis. 

The aforementioned features of whole saliva mean that it is crucial to carry out time- and resource-intensive pre-analytic steps before the actual detection and analysis of the target proteins that are of diagnostic significance. The most commonly used method for the pre-analytic preparation of whole saliva includes a freezing step for a time period that may range from a few hours to overnight or even longer. This has been reported to result in a reduction of saliva viscosity [23]. Before freezing, or upon thawing, a centrifugation step which can last up to 20 min, is conducted. This is carried out so that the agglomerates and other particles, like remnants of food, cells, or microorganisms [24], form a pellet and the supernatant can be used for further application-dependent analysis [5,17,18,25]. This method of pre-analytic processing of whole saliva is suitable, for example, in cases of central laboratories and clinics with much available space and infrastructure, and in cases when the time-to-result is not an issue. However, this reference method requires (i) several manual steps, (ii) long and careful sample processing by experienced personnel, as well as (iii) the availability and use of additional equipment, such as freezers and high-force centrifugation devices. Thus, it is not suitable for chair-side or PoC devices, as the latter would lose their principal benefits of rapid time-to-result and on-site use right after collection. 

One of the most important requirements for a saliva-based PoC or chair-side diagnostic system is the use of the sample matrix directly after collection, in order to circumvent manual pre-analytic processing, reduce hands-on time, test duration, and avoid the use of external equipment [26,27]. Microfluidic systems that automate salivary protein detection have been introduced as potential candidate systems for PoC analysis and research and development activities in the field have increased over the years [18]. Some microfluidic systems focus on the automation of protein detection itself but do not address sample pre-analytics. Other publications describe automated systems, however, the saliva is processed ex situ in these systems which make use of the above described freezing/thawing/centrifugation pre-treatment method [28,29]. Nie et al. describe a simplified pre-treatment for their system, however, their collection tube must be kept on ice, they centrifuge the whole saliva on an external device with 13,000 g for 20 min, and insert the supernatant into their cartridge [30]. Christodoulides et al. freeze their samples at −80 °C but do not report further processing of the samples [31]. Herr et al. freeze the saliva samples at −20 °C, thaw them, use a microcentrifuge for 3 min and, after a dilution step, insert the supernatant into their cartridge where a filter membrane and an enhancement system are then used to prepare the sample for further analysis [12]. Other systems use one or more external filters as pre-treatment of the whole saliva sample before it is inserted into the respective automated systems [21,32]. Including filter membranes in the pre-treatment process might be a less generic approach [2], as the potential unspecific adsorption on the membrane has to be investigated in depth for all target proteins. This can pose an additional challenge in case multiple target proteins are to be detected. Even a small loss of target proteins in membrane/filter systems can be crucial because one of the main challenges associated with using saliva as a sample is the low concentration of target biomarkers compared to blood.

Centrifugal microfluidic systems have some inherent advantages in that they do not require external connections to pumps or tubes, are closed systems, and can be compatible with monolithic manufacturing methods. Implementation of mechanical treatment for sample homogenization on such systems has been introduced and uses glass and/or magnetic beads in a microfluidic chamber to enhance the mechanical treatment [33,34] for cell lysis as a preparation for nucleic acid detection. However, to the best of our knowledge, such configurations have not yet been applied to protein detection using saliva as a matrix.

In our work, we present magnet-beating as a method for fully automated in situ pre-analytics of whole saliva in a centrifugal microfluidic platform. Samples are processed at room temperature, directly after collection, for downstream protein analysis. Three protein markers of periodontitis, a major oral disease affecting the tissue that surrounds and supports the teeth, were tested within this scope: matrix metalloproteinases MMP-8, MMP-9, and tissue inhibitor of metalloproteinases TIMP-1. Our method uses only a bar-shaped magnet (with no additional beads), actuated through a magnetic field and exerting mechanical forces on whole saliva inside a microfluidic chamber. No external steps or extra devices, apart from the PoC device itself, are necessary and no filter is used. This simplifies the integration and reduces the probability of unspecific binding. Characterization of the method included viscosity and biochemical measurements on the samples after magnet-beating and comparison with the reference method.

## 2. Materials and Methods 

### 2.1. Sample Collection

The saliva samples were collected from eight volunteer donors. All donors provided informed consent and the Ethics Committee of the University of Freiburg had no ethical or legal concerns regarding the data included in this work (request number: 10018/19). The number of samples was chosen in order to demonstrate the microfluidic proof-of-principle at a small scale, which would then allow us to continue further work towards full integration and subsequent clinical study, where more samples will be used. The donors were asked not to eat, drink (except water), smoke, nor chew any chewing gum within one hour prior to sample collection. The mouth was rinsed with water at least twice. The donors were given a simple, non-sterile sample container (polypropylene, 40 ml, Rotert, Germany) to collect the accumulating saliva without active stimulation of the saliva flow. The samples were divided into aliquots for further analysis using: (i) processing with the reference method, (ii) processing with magnet-beating, or (iii) untreated whole saliva. 

### 2.2. Reference Method

**Figure 1:**
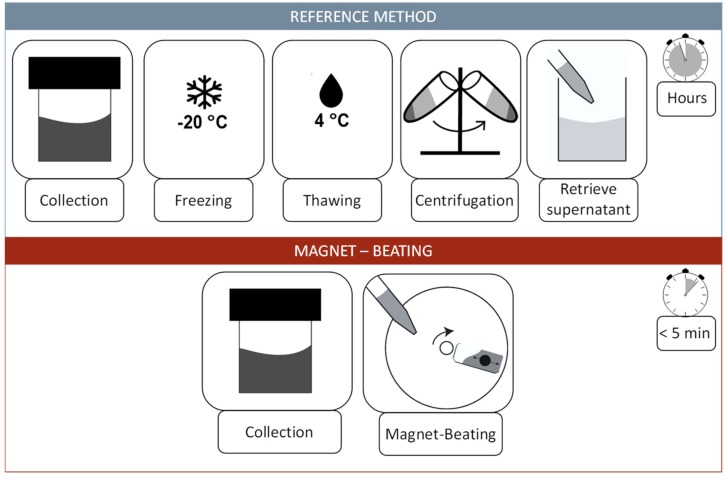
Top: Schematic description of the most widely used pre-analytic workflow (reference method) for whole saliva treatment for protein analysis. It includes the collection of the sample and a freezing step which can last a day or longer. The next steps are thawing at 4 °C and centrifugation to separate the pellet from the supernatant. The supernatant is then used for further protein analysis. Bottom: The magnet-beating workflow includes only the collection of the sample and pipetting onto the disk. The magnet-beating itself is completely automated and conducted at room temperature. The sample can be either processed further in situ (on disk) or ex situ (transferred to another device).

Collected samples were treated according to the reference method (Figure 1). The reference method was used on 500 µL whole saliva aliquots, involved freezing at −20 °C overnight, followed by a thawing step at 4 °C and centrifugation at 4 °C with 2000 g for 10 min (5415R, Eppendorf centrifuge, Eppendorf, Germany). The supernatant was pipetted and used for further analysis. 

### 2.3. Automated Centrifugal Microfluidic System

The centrifugal microfluidic disk cartridges were designed using SOLIDWORKS 17 (Dassault Systèmes, France) and fabricated by means of thermoforming of thin polycarbonate foils (thickness: 250 µm) as described by Focke et al. [35]. They were sealed with a piece of pressure-sensitive adhesive foil 9795R (3M, Maplewood, MN, USA) including a bar-shaped magnet (2 × 4 mm, Neodym, N48H Zinc, magnets4you, Germany) in the oval-shaped chamber. Then, 400 µL of whole saliva was pipetted into the chamber containing the bar-shaped magnet.

**Figure 2:**
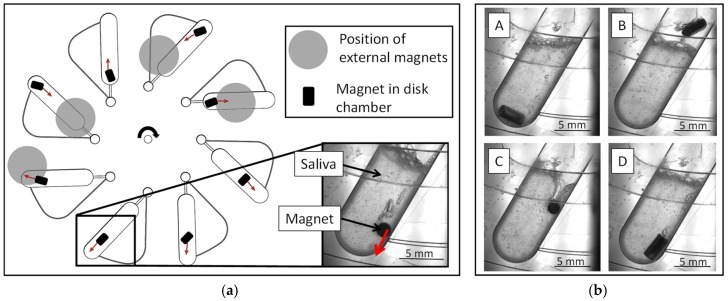
(**a**) Schematic illustration of the magnet-beating workflow on the disk, indicating the location of the external magnets which are mounted beneath the lid of the LabDisk Player [36]. The motion of the bar-shaped magnet under the influence of the magnetic and centrifugal forces and the orientation of its axis with respect to the chamber axis are illustrated in a simplified way (red arrows); (**b**) (**A**) The bar-shaped magnet is moved towards the radially outer edge of the chamber by the centrifugal force. (**B**) The force exerted by the external magnet moves the bar-shaped magnet in the chamber radially inwards through the saliva. (**C**,**D**) The bar-shaped magnet is moved radially outwards again by the now dominant centrifugal forces.

The disks were processed in a laboratory prototype LabDisk Player (QIAGEN Lake Constance, Germany), developed for processing thermoformed LabDisks with a precise motor and temperature control. Magnet-beating was conducted by rotating the disk at 15 Hz for 4 min below four permanent magnets mounted beneath the lid of the processing device [36] (Figure 2). Upon completion of the magnet-beating processing, the sample was pipetted out of the cartridge for further analysis. For real-time observation of the magnet-beating process, a version of the LabDisk Player, equipped with a stroboscopic function (BioFluidix, Germany) and a camera, was used.

### 2.4. Fluidic and Biochemical Characterization of Samples

The viscosity of whole saliva and water, as a reference, was measured with the rheometer Physica MCR101 (Anton Paar, Austria) using a conical plate CP25-1 applying a ramp from 50 to 3000 s^−1^ for the shear rate. 

To determine the total protein concentration of whole saliva, the Pierce BCA assay (Thermo Fisher Scientific, Waltham, MA, USA) was used, while the protocol of the assay was optimized for the read-out with a volume of 2 µL on the NanoDrop One (Thermo Fisher Scientific, Waltham, MA, USA). The samples were diluted twice with double-distilled water. The working reagent was mixed together with 50 parts reagent A and one part reagent B. Then, 20 µL working reagent and 5 µL standards or sample were mixed and incubated for at least 15 min at 37 °C. The total protein concentration of the sample was calculated with the help of a BSA (bovine serum albumin) standard. 

Enzyme-linked immunosorbent assay (ELISA) was performed with three salivary periodontal markers, namely MMP-8, MMP-9, and TIMP-1. The ELISA kits were provided by BioVendor (Czech Republic) and were used according to the supplier’s protocols, with sample dilutions as follows: 50-fold for MMP-8, 80-fold for MMP-9, and 250-fold for TIMP-1. The read-out was conducted on a microtiter plate reader (Spark M10, Tecan, Switzerland). The unknown concentrations of the samples were deduced from the standard curves using 4-parameter logistic fit (Supplementary Figure S1).

### 2.5. Design of Experiments (DoE)

Design of Experiments (DoE) for optimizing the system was created with Minitab 19 (Minitab GmbH, Germany) using a screening design with viscosity (at a constant shear rate 100 s^−1^) and total protein concentration as the output. Rotating frequencies between 5 and 15 Hz, magnet-beating durations between 1 and 4 min, sample volumes between 200 and 400 µL, and the presence (addition of 250 mg stainless steel beads with a diameter of 0.2 mm, Thermo Fisher Scientific, Waltham, MA, USA) or absence (only the bar-shaped magnet) of beads in the chamber were tested according to the screening design (Supplementary Table S1) by Minitab. The statistical evaluation in the form of a pareto-effect, variance analysis, and main effect was conducted with Minitab 19, too.

## 3. Results and Discussion

### 3.1. Magnet-Beating Operating Principle

Magnet-beating was developed for the automated pre-analytic processing of whole saliva for protein analysis. For magnet-beating, an oval-shaped chamber connected to a vent was designed on a microfluidic disk. The whole saliva is pipetted in the chamber and onto the disk that contains a bar-shaped magnet. External magnets (Figure 2a) are mounted beneath the lid of the processing device, the LabDisk Player, and induce magnetic forces onto the bar-shaped magnet on the disk (Figure 2b: A,B). Simultaneously, the rotation of the disk creates centrifugal forces which also affect the bar-shaped magnet. Depending on the location of the chamber beneath the external magnets, these centrifugal forces can be stronger than the magnetic forces (Figure 2b: C,D). The alternating dominance of centrifugal and magnetic forces leads to a rapid up- and downwards motion of the bar-shaped magnet, passing through the saliva. The repeated actuation of the bar-shaped magnet in the chamber through the saliva exerts shear forces on the latter resulting in sample homogenization and reduction of viscosity. As a result, the whole saliva becomes easily usable for subsequent protein analysis. 

### 3.2. Design of Experiments Analysis for Magnet-Beating Optimization

Since multiple experimental parameters are involved in this analysis, a DoE was performed in order to define the factors which are crucial for optimized performance. The considered input parameters which significantly influence the outcome of the pre-treatment of whole saliva with magnet-beating are: (i) the frequency of disk rotation; (ii) the duration of magnet-beating; (iii) the sample volume; and (iv) the inclusion or exclusion of beads in combination with the bar-shaped magnet. The first two are inherent parameters of our processing system (microfluidic disk). The sample volume was included in the DoE because the accurate pipetting of saliva has been found to be challenging in past experiments (Supplementary Figure S2) due to the non-Newtonian behavior of the whole saliva [37]. We included beads as a DoE parameter because they were efficiently used in cell lysis for PCR [34] and we wanted to see if this is also the case for whole saliva treatment and downstream protein analysis. For the evaluation of the system for the pre-analytic processing of whole saliva for protein analysis, we looked at two key output factors in the DoE, namely viscosity and total protein concentration. The former is an indicator of the behavior of liquid (Newtonian or non-Newtonian) and subsequently, its degree of handling efficiency by a microfluidic system, its mixing quality, etc. These properties have an impact on the downstream protein analysis. Total protein concentration can give an indication of the impact that magnet-beating may have on the protein content of the sample.

All of the above four input parameters were evaluated with a screening DoE. According to the DoE results, the magnet-beating settings were chosen to be the following: 400 µL sample volume treated at 15 Hz disk rotating frequency for a duration of 4 min and without the use of beads but only the bar-shaped magnet (more details in Appendix A and Supplementary Table S1).

### 3.3. Impact of Magnet-Beating on the Fluidic Behavior of Whole Saliva

**Figure 3:**
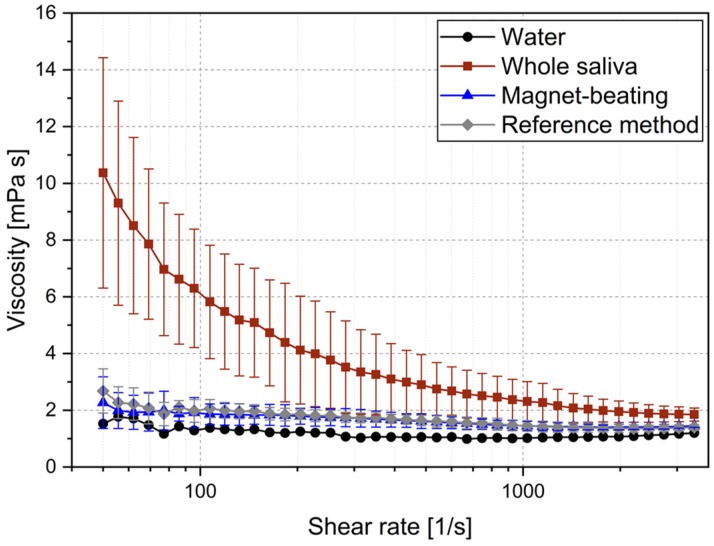
Viscosity of water as a reference for a Newtonian fluid, whole saliva (untreated) as a non-Newtonian fluid, whole saliva treated with magnet-beating, and whole saliva treated with the reference method. All measurements of the three latter cases were conducted on the same samples (N = 3).

Using the parameters defined by the DoE, the performance of the system was tested with regard to its impact on whole saliva viscosity. We compared the viscosity of three whole saliva samples: (i) treated with magnet-beating; (ii) treated according to the reference method; and (iii) without treatment. Such measurements were crucial in order to assess the impact of our system because whole saliva sample handling challenges are related strongly to viscosity [20]. Shear rates between 50 and 3000 s^−1^ were used to observe possible differences in viscosity behavior of the samples as shear forces changed (Figure 3). As seen in Table 1, at the low shear-rate of 50 s^−1^, the viscosity of treated saliva was between 3.8× and 4.5× lower (for reference and magnet-beating treatment respectively) than the viscosity of the untreated saliva. This is quite a substantial improvement in the fluidity of the matrix as it approached water-like behavior and its applicability in microfluidic systems therefore improved. At high shear rates of 3000 s^−1^, no significant difference in viscosity was found between the samples treated with the reference method and those which underwent magnet-beating. No difference was found either when they were compared to the untreated whole saliva (though they were still less viscous than the latter). Furthermore, the treatment of saliva (with either of the two methods) was shown to lower the standard deviation of the viscosity measurements at low shear rates.

Over the entire shear rate range (Figure 3), we observe a shear rate-dependent viscosity of untreated whole saliva, which confirmed its non-Newtonian behavior, as expected from literature [14]. This behavior is mainly attributed to the presence of glycoproteins (mucins), large molecular chains which can connect even further and thereby form networks [15,38,39]. The non-Newtonian behavior makes it difficult to meter defined amounts of whole saliva with low deviation (Supplementary Figure S2). However, after magnet-beating, the whole saliva viscosity became independent of shear rate, therefore exhibiting a Newtonian liquid behavior, just like it does after treatment with the reference method and similarly to water. This change in fluidic property of the sample matrix leads to its easy handling as well as efficient post-treatment mixing on the microfluidic disk (Supplementary Figure S3). Regarding the reference method, Schneyer indicated that the coagulate consists mainly of salivary mucoid after freezing and thawing and that viscosity is reduced [23]. Regarding the magnet-beating treatment, a possible explanation of its impact on the viscosity may be that the influence of mucine, which is mainly responsible for the viscosity of unstimulated saliva [16], is reduced either by breaking up the mucine network/agglomerations or by changing the tertiary structure of the mucine network. In any case, our proposed method is as efficient in this respect as the reference method and provides the desired results in a much shorter period of time and in an automated way. 

### 3.4. Impact of Magnet-Beating on Protein Biochemistry

Total protein concentration was evaluated using a BCA assay to measure whole saliva samples from four voluntary donors after magnet-beating compared to untreated whole saliva and the reference treatment method. Untreated whole saliva is difficult to pipette (Supplementary Figure S2) and although the pipetting was conducted carefully, a deviation of the whole saliva volume cannot be excluded. Interestingly, neither the reference method nor the magnet-beating method showed a significant negative impact on the total protein concentration of the treated samples. In fact, magnet-beating showed a higher total protein concentration for all measured samples (N = 4, for details see Supplementary Table S2). As the BCA assay detects peptide bonds, an increase of the total protein concentration means that the assay detects a higher number of peptide bonds. This may be a further indication that magnet-beating results in the disruption of the mucine network, potentially releasing proteins that previously formed a complex with the mucine network [40,41,42] and thereby enabling the detection of additional peptide bonds with the BCA assay [43].

**Figure 4:**
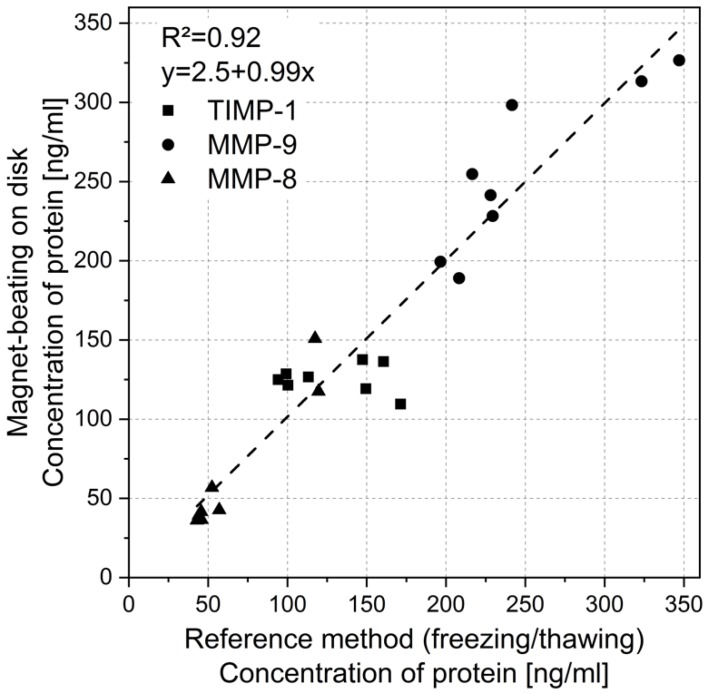
Protein concentration of the oral biomarkers MMP-8, MMP-9, and TIMP-1 obtained with ELISA after whole saliva processing using the reference method versus the magnet-beating method (N = 8).

The impact of magnet-beating as a pre-analytic treatment of whole saliva on the downstream analysis of specific salivary periodontal biomarkers (MMP-8, MMP-9, and TIMP-1) was tested by performing ELISA as this is the typical method used for protein marker immunoassays. Samples were collected from eight voluntary donors and separated into two 500 µL aliquots for processing with the reference method of freezing, thawing, and centrifugation, as well as with magnet-beating. ELISA was performed on both aliquots after pre-analytic processing. The ELISA results of magnet-beating showed a clear correlation with the results from the reference method with a slope of 0.99 of the linear fit and an R^2^ of 0.92 (Figure 4). 

This shows that the pre-analytic method of magnet-beating causes no reduction in protein concentration (for the selected periodontal protein markers) but also correlates excellently with the reference method. We presume that the ELISA performance on the mechanically-treated whole saliva correlates so well with the ELISA performance on the reference (non-mechanically) treated whole saliva due to impact on viscosity itself (since both treatment mechanisms also result in the same viscosity behaviors, Figure 3). We assume that the treatment of whole saliva (mechanical or not) influences factors that may be biochemically interfering with the ELISA. It may have an impact on the glycoprotein mucine (20% of the proteins found in saliva [40]) by presumably distorting its network formation and complex-forming with other salivary proteins [40,41,42], thereby enabling biochemical interactions of these (target-biomarker) molecules and detection with ELISA. These assumptions are derived from experimental observations in combination with information available in the literature [40,41,42]. They could be elaborated and generalized by dedicated investigation of the actual biochemical interactions between many target molecules and mucins, which was, however, outside the scope of this work. In any case, if magnet-beating is able to influence the gel matrix of the glycoproteins [39], then errors in concentration measurements due to proteins trapped in the complexes can be reduced, thereby enabling magnet-beating pre-analytic processing to substitute the time-consuming and manual reference method.

## 4. Conclusions

In this paper we presented a method for pre-analytic processing of whole saliva based on mechanical treatment via magnet-beating and demonstrated the proof-of-principle of its microfluidic integration that enables us to proceed with higher degree of analytical integration in the future. We implemented the method on a centrifugal microfluidic cartridge, but in principle it can be applied on any platform that can actuate a bar-shaped magnet in a chamber. We used DoE for parametric characterization of the system, which resulted in optimized conditions of using only the bar-shaped magnet (and no additional beads), 400 µL sample volume in a microfluidic disk rotating at 15 Hz for 4 min. Both the reference method and the magnet-beating method had a similar, positive effect on the viscosity of the whole saliva, a key parameter for efficient handling on a microfluidic system. Furthermore, ELISA results for three major periodontal biomarkers in magnet-beating treated whole saliva samples showed excellent correlation with the results acquired using the reference treatment method. This indicates that the magnet-beating method is suitable as a pre-analytic process for whole saliva. In addition, the treatment duration was reduced from hours (under reference method) to less than 5 min. Consequently, achieving the same fluidic and biochemical impact on whole saliva with the magnet-beating method as with the reference method, and doing so in a much shorter period of time and with a chair-side/PoC-compatible platform, paves the way for the integration of the magnet-beating into a fully-automated sample-to-answer saliva-based biomarker detection workflow, performed with minimal hands-on steps and user intervention at room temperature. 

## Figures and Tables

**Table 1:** Representative measurements of viscosity (N = 3) of untreated whole saliva, and treated with reference and magnet-beating methods at low and high shear rates. The full shear rate range can be seen in Figure 3.

Treatment	Shear rate [1/s]	Mean viscosity [mPa s]	Standard deviation [mPa s]
None	50	10.4	4.1
	3000	1.9	0.3
Reference method	50	2.7	0.8
	3000	1.4	0.1
Magnet-beating	50	2.3	0.9
	3000	1.4	0.1

**Figure A1:**
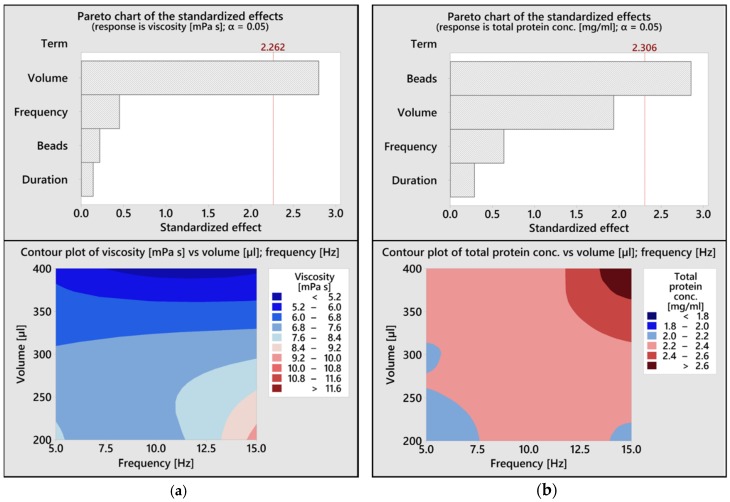
(**a**) The upper graph shows the result of the pareto analysis conducted with Minitab 19 using the viscosity as an output. The lower contour plot shows the relationship of the two input factors volume and disk rotating frequency with the output factor viscosity; (**b**) The upper graph shows the result of the pareto analysis using the total protein concentration as an output. The lower contour plot shows the relationship of the two input factors volume and disk rotating frequency with the output factor total protein concentration.

## Supplementary Materials

The following are available online at https://www.mdpi.com/2072-666X/10/12/833/s1, Figure S1: Standard curves from the ELISA measurements, Figure S2: Measurement of whole saliva volumes, Figure S3: Mixing on disk, Table S1: Screening design for the DoE, Table S2: Total protein concentration of untreated and treated whole saliva samples.

## Appendix A. Details on the Design of Experiments Analysis

The pareto chart for viscosity as a response shows that viscosity was mainly affected by the sample volume which is introduced into the chamber (Figure A1a). The contour plot shows the relationship of the two input factors volume and frequency with the output factor viscosity. To achieve a desired low viscosity, a high sample volume of 400 µL should be chosen. The frequency shows only a minimal impact in that regard. The pareto chart for the total protein concentration as response, which was analyzed using a BCA assay, shows that only the presence of 250 mg beads has a significant impact (0.2 mm stainless steel beads were chosen after an evaluation of different sized beads; data not shown) (Figure A1b). The data of the DoE showed a lower mean of the total protein concentration with 2.1 mg/mL for all runs conducted with beads, compared to the mean of 2.5 mg/mL for all runs conducted with only the magnet. Those data showed clearly that the presence of only the bar-shaped magnet with no additional beads is sufficient for the pre-treatment of saliva. Using only a magnet is advantageous from disk preparation perspective, too, because avoiding to weigh the beads before every measurement reduces the number of steps associated with disk preparation and the handling challenges during sealing due to electrostatically-induced bead misplacement. Considering the contour plot for the total protein concentration, it can be seen that the combination of a high sample volume with a high disk rotating frequency results in the highest protein yield.
