# Supplementary material for: Automated Pre-Analytic Processing of Whole Saliva Using Magnet-Beating for Point-of-Care Protein Biomarker Analysis
Source: Micromachines (Basel). 2019 Nov 30;10(12):833. doi: 10.3390/mi10120833 (PMC6952956; doi:10.3390/mi10120833)
Supplement: Supplementary file 1 [file micromachines-10-00833-s001.pdf]

micromachines

Supplementary Materials: Automated Pre-Analytic Processing of Whole Saliva Using Magnet-Beating for Point-of-Care Protein Biomarker Analysis

Benita Johannsen [1,*], Lara Müller [1], Desirée Baumgartner [1,2], Lena Karkossa [1], Susanna M. Früh [1,2], Nagihan Bostanci [3], Michal Karpíšek [4,5], Roland Zengerle [1,2], Nils Paust [1,2] and Konstantinos Mitsakakis [1,2,*]

1 Hahn-Schickard, Georges-Koehler-Allee 103, 79110 Freiburg, Germany; lara-mueller95@gmx.de (L.M.); Desiree.Baumgartner@imtek.uni-freiburg.de (D.B.); karkossal@online.de (L.K.); Susanna.Frueh@Hahn-Schickard.de (S.M.F.); Roland.Zengerle@hahn-schickard.de (R.Z.); Nils.Paust@Hahn-Schickard.de (N.P.)

2 Laboratory for MEMS Applications, IMTEK — Department of Microsystems Engineering, University of Freiburg, Georges-Koehler-Allee 103, 79110 Freiburg, Germany

3 Section of Periodontology and Dental Prevention, Division of Oral Diseases, Department of Dental Medicine, Karolinska Institutet, Alfred Nobels Allé 8, 14104 Huddinge, Stockholm, Sweden; nagihan.bostanci@ki.se

4 BioVendor — Laboratorní medicína a.s., Research & Diagnostic Products Division, Karasek 1767/1, Reckovice, 62100 Brno, Czech Republic; karpisek@biovendor.com

5 University of Veterinary and Pharmaceutical Sciences Brno, Faculty of Pharmacy, Palackeho trida 1946/1, 61242 Brno, Czech Republic

* Correspondences: Benita.Johannsen@Hahn-Schickard.de (B.J.); Konstantinos.Mitsakakis@Hahn-Schickard.de (K.M.); Tel.: +49-761-203-7252 (B.J.); +49-761-203-73252 (K.M.)

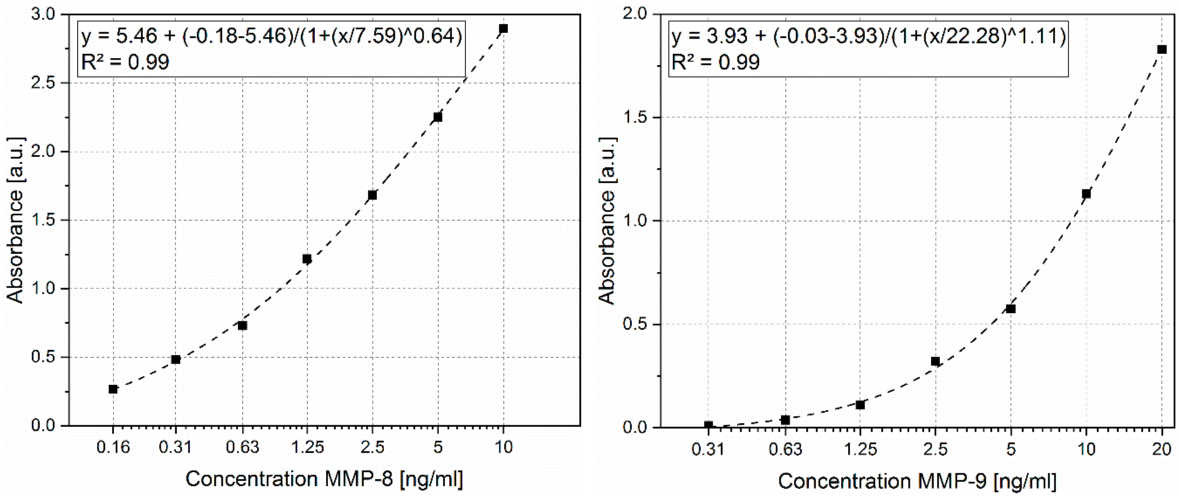

(**a**) (**b**)

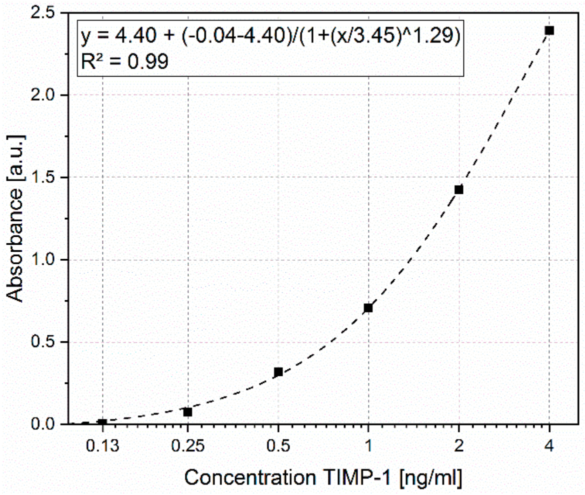

(**c**)

Figure S1. Standard curves from the ELISA measurements of the three salivary biomarkers MMP-8 (**a**), MMP-9 (**b**), and TIMP-1 (**c**). The standard curves follow a logistic 4-parameter fit of the form y = A2 + (A1 − A2)/(1 + (x/X0)^p).

Table S1. Screening design for the Design of Experiments (DoE) in Minitab.

RunOrder	Beads (Yes/No)	Volume (µL)	Frequency (Hz)	Duration (min)	Viscosity (mPa·s)	Total Protein Concentration (mg/mL)
1	YES	300	10	2.5	8.3	1.9
2	YES	200	5	4	6.4	2.2
3	YES	200	15	2.5	6.9	1.8
4	NO	200	10	1	7.2	2.2
5	YES	200	5	1	9.1	2.0
6	NO	300	10	2.5	6.0	2.8
7	YES	300	15	1	7.5	Error in System
8	NO	300	5	4	6.9	2.2
9	YES	400	10	4	4.9	2.3
10	YES	400	5	1	6.1	2.3
11	NO	400	5	2.5	5.5	2.5
12	NO	200	15	4	12.3	2.6
13	NO	400	15	4	4.5	2.7
14	NO	400	15	1	5.6	2.7

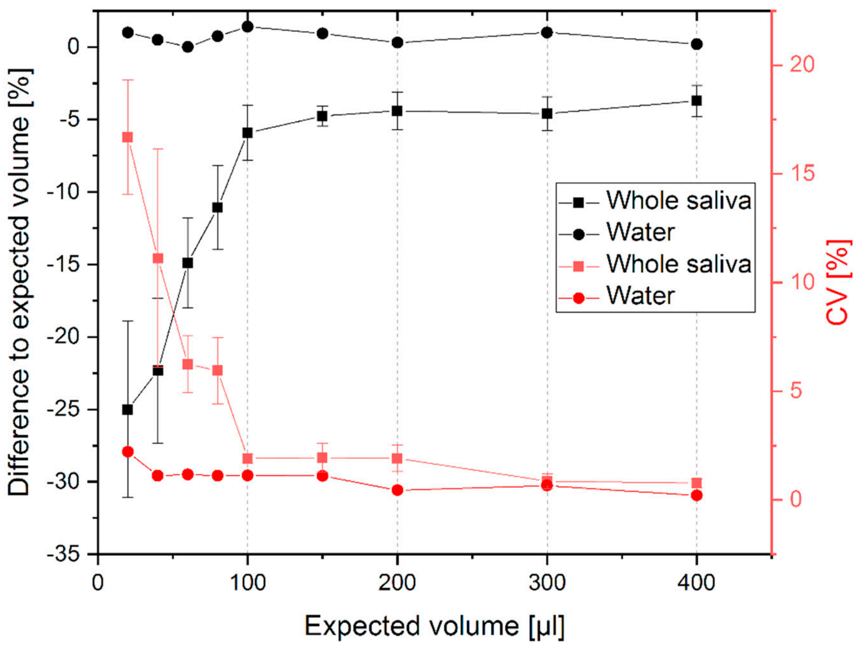

Figure S2. Measurement of the volume of freshly collected saliva samples by weighing the pipetted amount of whole saliva. Three different samples were provided by voluntary donors. All samples were pipetted 5 times. The deviation of the pipetted from the expected volume is shown in the left Y-axis. The variation of the repeated pipetting is shown as a CV in the right Y-axis. Water was used as a reference.

Table S2. Total protein concentration measured with the BCA assay. The same samples were treated either with magnet-beating or with the reference method and the derived total protein concentration was compared to the total protein concentration of the untreated whole saliva samples.

	Total Protein Concentration [mg/mL] Measured with BCA Assay		
**Sample**	**Whole Saliva**	**Magnet-beating**	**Reference Method**
1	3.88	4.26	3.32
2	3.35	5.14	3.47
3	2.20	2.40	2.30
4	3.22	3.93	4.18

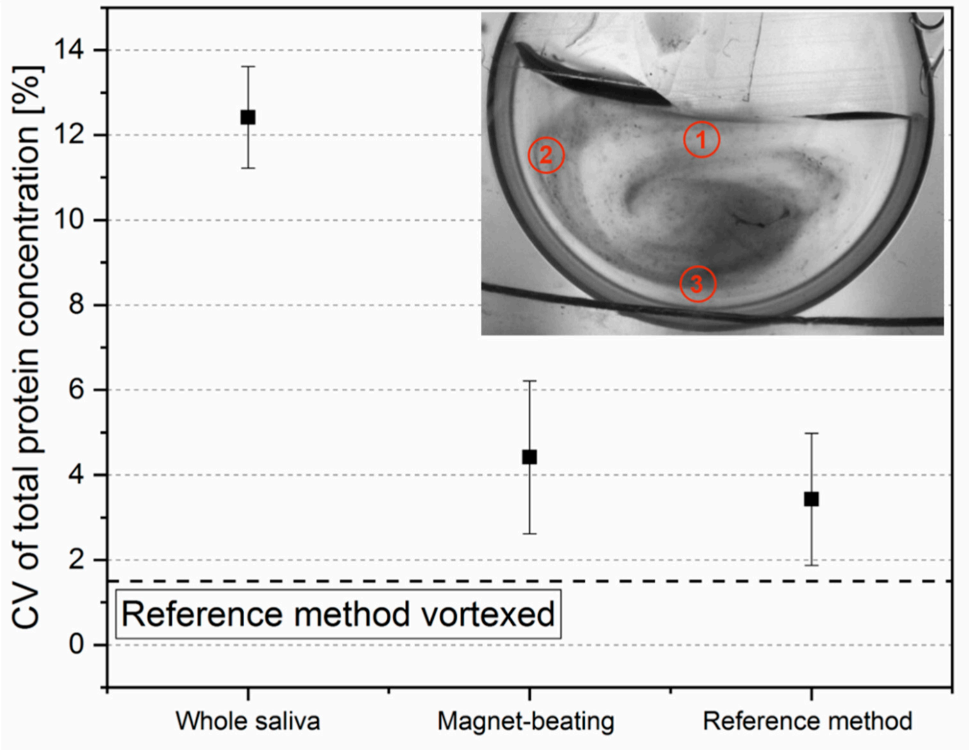

Figure S3. Batch-mode mixing (DOI: 10.1039/B418253G) was conducted on disk after the magnet-beating. Three whole saliva samples (fresh, treated with magnet-beating, and treated with the reference method) were mixed with distilled water (1:10) on disk in a total volume of 70 µL. After the mixing on disk (with min/max −10/10 Hz, acceleration 40 Hz/s) for 1 min, three representative samples of 5 µL were pipetted out of the mixing chamber from three different positions (see red numbers). The total protein concentration was evaluated using a BCA assay to gain information on the homogeneity of the mixing. The CV of the total protein concentration between the three representative samples within one mixing chamber is shown and is approximately 3× lower for the treated than the untreated whole saliva. As a reference, vortex mixing of untreated whole saliva in a tube was used (dashed line), where consequently also three different positions in the tube were tested after mixing.

© 2019 by the authors. Submitted for possible open access publication under the terms and conditions of the Creative Commons Attribution (CC BY) license (http://creativecommons.org/licenses/by/4.0/).
